# Synthesis of the Novel Type of Bimodal Ceramic Nanowires from Polymer and Composite Fibrous Mats

**DOI:** 10.3390/nano8030179

**Published:** 2018-03-20

**Authors:** Tomasz Tański, Wiktor Matysiak

**Affiliations:** Institute of Engineering Materials and Biomaterials, Silesian University of Technology, Konarskiego 18a, 44-100 Gliwice, Poland

**Keywords:** SiO_2_ nanowires, TiO_2_ nanowires, bimodal nanowires, electrospinning, Zol-gel method, optical properties

## Abstract

The purpose of this paper was to produce SiO_2_ and TiO_2_ nanowires via the electrospinning process from a polyvinylpyrrolidone (PVP)/Tetraethyl orthosilicate (TEOS)/Titanium (IV) butoxide (TNBT)/dimethylformamide (DMF) and ethanol (EtOH) solution. The as-obtained nanofibers were calcined at temperatures ranging from 400 °C to 600 °C in order to remove the organic phase. The one-dimensional ceramic nanostructures were studied using a scanning electron microscope (SEM) and a transmission electron microscope (TEM) to analyze the influence of the used temperature on the morphology and structures of the obtained ceramic nanomaterials. In order to examine the chemical structure of the nanowires, energy dispersive spectrometry (EDX) and Fourier-Transform Infrared spectroscopy (FTIR) were used. The optical property analysis was performed on the basis of UV-Vis spectra of absorbance as a function of the wavelength. Using the modified Swanepoel method, which the authors proposed and the recorded absorbance spectra allowed to determine the banded refractive index *n*, real *n*′ and imaginary *k* part of the refractive index as a function of the wavelength, complex dielectric permeability *ε*, and real and imaginary part *ε_r_* and *ε_i_* of the dielectric permeability as a function of the radiation energy of the produced ceramic nanowires.

## 1. Introduction

**Figure 1:**
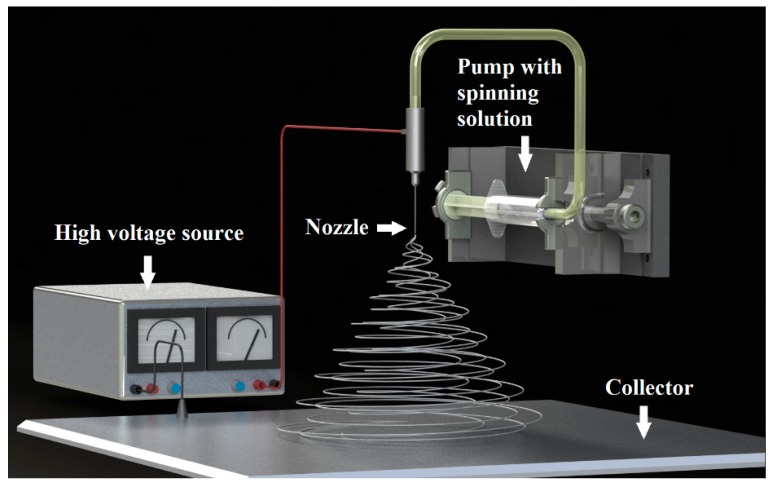
The scheme of the preparation of one-dimensional nanostructures by electrospinning.

Over the last fifteen years, one-dimensional structures of simple oxides particularly titanium oxide (TiO_2_) [1,2,3,4] and silicon oxide (SiO_2_) have been particularly popular in the field of both scientific research and expected wide application possibilities [5,6,7,8]. Unlike other zero-dimensional, two-dimensional, or three-dimensional nanostructures, nanowires have two limited quantum directions resulting from their nanometer diameter due to which electrons can easily move in a precisely defined direction, which is determined by the length of a single nanowire. This allows for the use of this structure in elements in which the main challenge is to conduct electricity excluding the tunneling transition. In addition, due to very high energy densities occurring in single-dimensional oxide nanomaterials resulting from the nanometric diameters of individual nanowires, these materials exhibit extremely different and better optical, magnetic, and electrical properties in abducting their counterparts in the micrometer scale [9]. To date, many techniques have been developed for producing nanowires and ceramic nanofibers. The most commonly used ones are physical vapor deposition (PVD), chemical vapor deposition (CVD), and thermal decomposition in which the growth of nanostructures is caused by vapor-liquid-solid (VLS) or solid-liquid-solid (SLS) mechanisms [10,11,12,13,14,15]. These methods allow to fully control the morphology of one-dimensional nanomaterials, but the production costs when using these methods are too high. This makes it unprofitable to use them to produce nanowires and oxide nanofibers on an industrial scale. The method of obtaining one-dimensional ceramic nanostructures based on the combination of sol-gel technology and spinning of the prepared sol in a strong electrostatic field is becoming more and more valued [16,17,18,19,20,21,22,23]. This process does not require complicated operations and expensive, technically advanced equipment. In addition, it is characterized by low production costs combined with the possibility of producing nanostructures on an industrial scale. In addition, by selecting the electrospinning process parameters such as the distance and voltage between the nozzle and collector as well as the spinning solution feed rate through the device pump, (see Figure 1) as well as the spinning solution parameters such as conductivity, viscosity, and precursor concentration and the conditions of the calcination process in the subsequent production stage of one-dimensional Ceramic nanostructures, it is possible to obtain nanowires and ceramic nanotubes characterized by a fully controlled and defined morphology. The ceramic nanostructures obtained in this way can be a more effective and more economical alternative to nanowires produced using conventional methods, which are increasingly used to build a new generation of photovoltaic cells, filters, or sensors.

So far, dye photovoltaic cells built on the basis of electrodes consisting of titanium dioxide nanostructures achieve the highest efficiency in converting solar energy into electricity-up to 11% on a laboratory scale. TiO_2_ is the ideal material for this type of application due to its physical properties such as the energy gap width of 3–3.4 eV, the refractive index at 2–2.5, the electric permittivity value of 8.5, and the ability for the formation of crystalline structures such as rutile, anatase, and brookite [24]. One-dimensional nanostructures of titanium oxide have already found their application as materials for photo-electrochemical water splitting [25] and oxidation [26] as well as the base for composite materials used for environmental purification [27] and photo-electrochemical hydrogen generation [28]. In turn, as demonstrated by Nishikawa et al., at room temperature silicon oxide is characterized by six photoluminescent bands in the energy range from 1.9 to 4.3 eV due to excitations induced by a 7.9 eV excimer laser [29]. Radiation emitted through amorphous SiO_2_ nanowires corresponding to visible light with a blue and green color allows the use of this type of nanostructures as novel green-emitting phosphor [30] or materials for the construction of full-color display [31]. In addition, stable phase change materials made on the basis of one-dimensional SiO_2_ nanostructures obtained using the sol-gel method and electrospinning showed high efficiency of heat energy collection and storage [32]. Additionally, nanofibrous SiO_2_ mats as well as composite materials produced with their participation are successfully used to build innovative, flexible lithium-ion batteries [33] and water filters, which allows the adsorption of toxic cationic and anionic dyes. This is possible due to the high porosity and specific surface of the nano-structured silica [34].

The purpose of the presented work is to create modern ceramic nanostructures in the form of bimodal nanowires with titanium oxide and silicon oxide matrices containing nanoparticles of the same oxide as the matrix as well as to determine the impact of the applied nanoparticles and calcination temperatures on the morphology, structure, and optical properties of the obtained bimodal nanowires of SiO_2_/SiO_2_ and TiO_2_/TiO_2_.

## 2. Materials and Methods

### 2.1. Materials

To prepare spinning solutions and produce one-dimensional nanostructures, we used poly (vinylpyrrolidone) (PVP, 99% pure, *M*_w_ = 1,300,000 g/mole), ethanol (EtOH, 99.8% purity), acetic acid (AcOH, 99.8% purity), and titanium (IV) butoxide (TNBT, purity 98%), tetraethoxysilane (TEOS, purity 99%), and deionized water (H2OEthyl alcohol was supplied by Avantor Performance Materials Poland (Gliwice, Poland). Other reagents and polymers were purchased from Sigma-Aldrich (Poznan, Poland). In order to obtain bimodal SiO_2_/SiO_2_ nanostructures, SiO_2_ nanoparticles were additionally used at the stage of the spinning solution preparation while, in the case of the TiO_2_/TiO_2_ bimodal nanostructures, the nanofillers were TiO_2_ nanoparticles. The applied nanopowders were characterized by the author in the work [35].

### 2.2. Methods

In the first step of preparing the spinning solutions to obtain SiO_2_ nanowires and bimodal SiO_2_/SiO_2_ nanowires, two identical 10% (*w*/*w*) of polymer/ethanol mixtures were produced by adding 3.825 g of PVP to 50 mL of EtOH, where, for one of the solutions, before adding the polymer to ethanol, a metered amount of SiO_2_ nanoparticles of 1.095 g, which corresponds to 25% of the weight concentration of nanoparticles relative to the polymer mass, was added. This was followed by sonication for 1 h to break up the agglomerates of the applied nano powder. Two milliliters of H_2_O were added to the mixture and then everything was stirred in magnetic stirrers for 60 min. In the next step, a mixture of 4 mL of TEOS with 4.66 mL of AcOH was added to each solution and it was further stirred for 24 h. In the case of the preparation of spinning solutions to obtain TiO_2_ nanowires and bimodal TiO_2_/TiO_2_ nanowires, two equal 10% (by weight) polymer blends in ethanol were also prepared by adding 3.825 g of PVP to 50 mL of EtOH. In the case of one of the solutions, before adding the polymer to ethanol, a measured amount of 1.095 g of TiO_2_, which correspond to 25% of the weight concentration of nanoparticles relative to the weight of the polymer nanoparticles, was added and the whole was sonicated for 1 h to break up the agglomerates of the used reinforcing phase. The prepared mixtures were mixed in magnetic stirrers for 60 min. In the next step, a mixture of 4 mL of TNBT with 4.66 mL of AcOH was added to each solution and further stirred for 24 h.

Immediately after the mixing was completed, the solutions were placed in the device’s pump and subjected to electrospinning. The nanofibers were obtained with the use of the FLOW-Nanotechnology Solutions Electrospinner 2.2.0–500 device (Yflow, Malaga, Spain) using the following process parameters: solution flow rate of 2.5 mL/h, potential difference between electrodes of 20 kV, and distance between electrodes of 17.5 cm for solutions containing SiO_2_ and 15 cm in the case of TiO_2_.

Immediately after production, the fibrous mats were allowed to dry at room temperature and then they were subjected to the calcination process in a HT-2100-G-Vac-Graphit-Special high-temperature vacuum furnace oven (Linn, Bad Frankenhausen, Germany) at temperatures of 400 °C, 500 °C, and 600 °C in an air atmosphere for a time of 3 h. In all cases, the heating speed was 10 °C/min and, after heating, the samples remained in the furnace until it cooled down.

In order to analyze the morphology and structure of the produced one-dimensional ceramic nanostructures, a high-resolution transmission electron microscope TITAN 80–300 from FEI (Hillsboro, OR, USA) was used, which enabled imaging in transmission and scanning-transmission modes by using light and dark fields (BF, DF), HAADF detector, and filtration energy in particular with the use of analytical microscopy in nano areas in STEM mode.

In addition, single-dimensional ceramic nanostructures were quantitatively and qualitatively analyzed using Fourier-Transform Infrared spectroscopy (FTIR) (Nicolet™ iS™ 50 FTIR Spectrometer, Thermo Fisher Scientific, Waltham, MA, USA) as well as X-ray EDX microanalysis (Trident XM4, EDAX, Weiterstadt, Germany) and surface topography imaging using a Zeiss Supra 35 scanning electron microscope (Zeiss, Oberkochen, Germany) with an X-ray Trident XM4 spectrometer provided by EDAX. Based on the taken SEM images, the diameters of one hundred randomly selected nanowires were measured using the Digital Micrograph program (2.32.888.0 version, GATAN, Pleasanton, CA, USA). Then their average value and chemical composition were determined based on EDX spectra.

To test the optical properties of TiO_2_ and SiO_2_ nanowires as well as bimodal TiO_2_/TiO_2_ and SiO_2_/SiO_2_ nanowires, they were applied to silicon substrates and then they were subjected to UV-Vis spectroscopic analysis. Measurements of the absorbance of the obtained materials as a function of the length of electromagnetic radiation incident on the sample were carried out using a Thermo-Scientific UV/VIS Evolution 220 spectrophotometer (Thermo Fisher Scientific, Waltham, MA, USA). Then, based on absorption spectra, the values of the band gap width, complex refractive index *n*′, refractive index *n* as a function of the wavelength, extinction coefficient *k* as a function of the wavelength, complex electrical constant, and real and imaginary part of the etheric constant *ε_r_* and *ε_i_* as a function of the wavelength were all calculated.

### 2.3. Theory

Electromagnetic radiation falling on any material medium can penetrate, reflect, or be absorbed by it when the energy carried by given radiation quanta is equal to the energy difference between the energy states of the atoms of the studied medium. The degree of the reflection of electromagnetic radiation from a sample is determined by the so-called reflectance, which is defined as the ratio of the reflected beam power and the beam of the material falling on a given medium. Transmittance, on the other hand, is defined as the ratio of the beam power after passing through a given center to the power of the beam falling on it. Starting from the Lambert-Beer law and the relationship between absorbance (*A*) and transmittance (*T*), one obtains Equation (1).

(1) $$A=\log\frac{I_{0}}{I}=-\log\left(T\right)$$

Using the recorded absorbance spectra as a function of length and the above equation, it is possible to determine transmittance as a function of the wavelength, which is observed in Equation (2).

(2) $$T\left(\lambda \right)=10^{-A\left(\lambda \right)}$$

Based on the above dependence and equality:

(3) $${\left\{h\nu \left[\ln\left(\frac{1}{T\left(\lambda \right)}\right)\right]\right\}}^{2}={\left[\alpha \left(\lambda \right)h\nu \right]}^{2}$$

where *h* is the Planck constant and ν is the frequency of incident radiation per sample. Using Equation (3), we define Equation (4) and the relationship between the absorption coefficient and the extinction coefficient.

(4) $$\alpha \left(\lambda \right)=\ln\left(\frac{1}{T\left(\lambda \right)}\right)$$

Equation (5) is calculated by using Equation (4) and the relationship between the absorption coefficient and the extinction coefficient.

(5) $$\alpha \left(\lambda \right)=\frac{4\pi k\left(\lambda \right)}{\lambda }$$

An expression is obtained for the extinction coefficient as a function of the wavelength.

(6) $$k\left(\lambda \right)=\frac{1}{4\pi }\lambda \ln\frac{1}{T\left(\lambda \right)}$$

**Figure 2:**
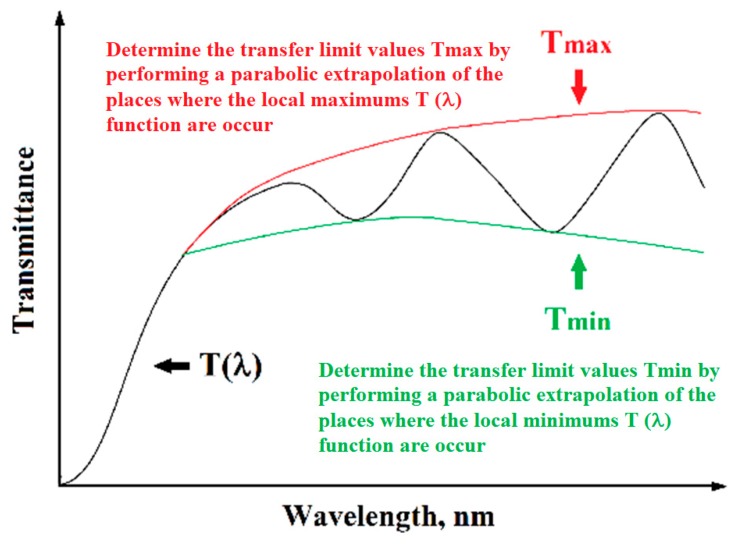
Schematic spectrum of transmittance as a function of the wavelength of a thin layer on a transparent substrate with visible local interference maxima and minima.

By plotting the determined dependence of transmittance (2) as a function of the wavelength of radiation incident on the sample, it can be seen that the spectrum is characterized by interference occurring at higher transmittance values (see Figure 2). The location of emerging interference maxima and minima of the *T*(*λ*) function is related to the actual part of the complex refractive index.

(7) $$n^{\prime }=n+ik$$

where *n*—the real part of the refractive index; *i* and *k*—is the extinction coefficient.

Using the R. Swanepoel method [36], which is limited to application only for thin layers deposited on a transparent substrate whose thickness is much greater than the thickness of the layer being tested, this condition is met for samples examined by the authors of this monograph. Based on the determined spectrum *T*(*λ*), the next step is to determine the transfer limit values *T_max_* and *T_min_* by performing a parabolic extrapolation of the places where the maximum and minimum queues occur (see Figure 2). After determining the limit values *T_max_* and *T_min_* using the dependence, Equation (8) is calculated below.

(8) n′={2npTmax−TminTmaxTmin+np2+12+[(2npTmax−TminTmaxTmin+np2+12)2−np2]12}12

where *n_p_*—is the refractive index of the substrate used, which includes the determined complex refractive index of the studied one-dimensional oxide nanostructures.

In addition, based on the dependencies:

(9) n′=ε′12

A complex dielectric permittivity coefficient can be determined, which takes the form of Equation (10).

(10) ε′=2npTmax−TminTmaxTmin+np2+12+[(2npTmax−TminTmaxTmin+np2+12)2−np2]12

Based on the derived dependence of the extinction coefficient as a function of the wavelength of electromagnetic radiation incident on the sample *k*(*λ*) and the determined reflection spectrum *R*(*λ*), Equation (11) is determined.

(11) A(λ)+T(λ)+R(λ)=1

The real part of the refractive index of the tested one-dimensional materials can be determined. The real part of the refractive index takes the form of Equation (12).

(12) n(λ)={4R(λ)[R(λ)−1]2−[14πλln1T(λ)]2}12−R(λ)+1R(λ)−1

Using the relationship between the real *n*(*λ*) and the imaginary *k*(*λ*) part of the refractive index and complex dielectric permeability *ε_r_*(*λ*), *ε_i_*(*λ*) has the form below.

(13) $$\varepsilon_{r}\left(\lambda \right)=n{\left(\lambda \right)}^{2}-k{\left(\lambda \right)}^{2}$$

(14) $$\varepsilon_{i}\left(\lambda \right)=2n\left(\lambda \right)k\left(\lambda \right)$$

The following equations describing the dependence between real *ε_r_*(*λ*) and imaginary *ε_i_*(*λ*) part of complex dielectric permittivity were obtained below.

(15) εr(λ)={{4R(λ)[R(λ)−1]2−[14πλln1T(λ)]2}12−R(λ)+1R(λ)−1}2−[14πλln1T(λ)]2

(16) εi(λ)=2{{4R(λ)[R(λ)−1]2−[14πλln1T(λ)]2}12−R(λ)+1R(λ)−1}[14πλln1T(λ)]

Based on the dependence of the absorption coefficient of the tested material as a function of the energy of incident radiation on its surface, which has the form below [37,38,39].

(17) $$\alpha h\nu =A{\left(h\nu -E_{g}\right)}^{\rho }$$

where, *h*—the Planck constant, is the frequency of electromagnetic radiation, *E_g_*—is the width of the energy gap of the tested material, *A*—it is a constant dependent on the probability of electron transitions, the values of the energy gap width of the produced one-dimensional oxide nanomaterials can be determined. In the case of coefficient *ρ*, various values, such as 1/2 and 3/2, are given for the available and the inaccessible direct inter-band transitions, and 2 and 3 for the successive and the forbidden intermediate transitions. However, the best results were achieved using an exponent equal to ½ and such was assumed in this work. To determine the band width of the produced one-dimensional ceramic nanowires, the absorbance spectra obtained by means of the UV-Vis spectroscope were used as a function of the wavelength of incident per sample. In order to eliminate the influence of the substrate on which the layers of the produced nanowires were located, the absorption spectrum was measured for the substrate itself and, according to the law of absorption additivity, they were subtracted from the spectrum obtained for the layer of nanowires deposited on the substrate. For the spectra obtained in this way, using Equation (2), the spectra of the dependence of transmittance as a function of the wavelength were determined for all generated nanomaterials. After transformation, Equation (17) takes the following form.

(18) $${\left[h\nu \ln\left(\frac{1}{10^{-ABS}}\right)\right]}^{2}=B\left(h\nu -E_{g}\right)$$

where B is a constant dependent on the probability of electron transitions divided by the thickness of the examined layer. Then, dependencies ${\left[h\nu \;\ln\left(\frac{1}{10^{-ABS}}\right)\right]}^{2}$ were plotted in the energy function of radiation quanta for all generated nanomaterials and linear functions were adjusted to straight sections of graphs with the largest directional coefficients of a straight line. Zero sites were determined by calculating the absolute value of the ratio of free expressions to the directional coefficients of matched lines (|b/a|) and corresponded to the width of energy gaps in the studied ceramic nanowires.

## 3. Results and Discussion

### 3.1. TEM Analysis

**Figure 3:**
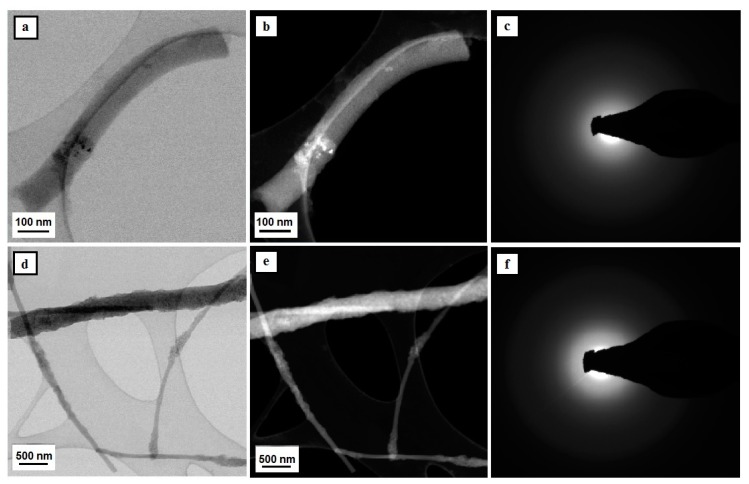
TEM images of the tested nanostructures – SiO_2_ nanowires comprising a matrix for SiO_2_/SiO_2_ NPs bimodal nanowires (**a**,**b**,**d**,**e**) along with diffraction images obtained with the use of analytical microscopy in nano areas in STEM mode and solved electronograms for nanoparticles and nanowires of silicon oxide (**c**,**f**).

**Figure 4:**
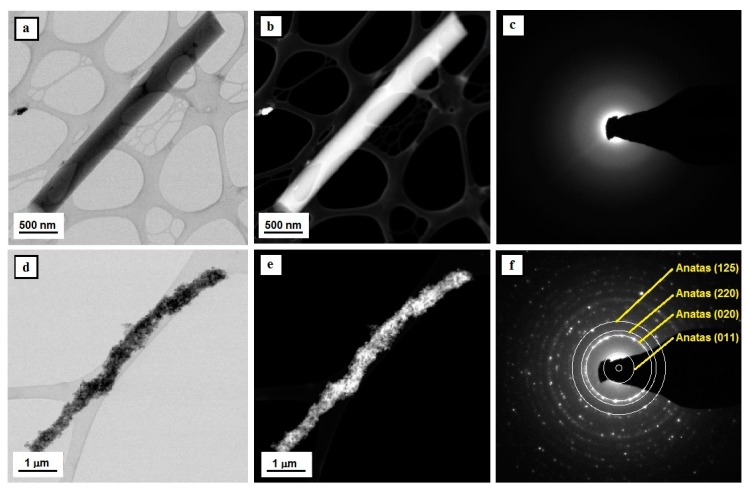
TEM images of the tested nanostructures—Tio_2_ nanowires comprising a matrix for TiO_2_/TiO_2_ NPs bimodal nanowires (**a**,**b**,**d**,**e**) along with diffraction images obtained with the use of analytical microscopy in nano areas in STEM mode and solved electronograms for nanoparticles and nanowires of titanium oxide (**c**,**f**).

The analysis of the produced one-dimensional nanostructures, which was conducted using a high-resolution transmission electron microscope, has unambiguously confirmed that the use of a combination of the sol-gel method and electrospinning from solutions of PVP/TEOS/EtOH/AcOH, PVP/TEOS/EtOH/AcOH/SiO_2_ NPs, and PVP/TNBT/EtOH/AcOH, PVP/TNBT/EtOH/AcOH/TiO_2_ NPs makes it possible to produce appropriately amorphous silicon oxide and titanium oxide nanowires as well as hybrid SiO_2_/SiO_2_ NPs and TiO_2_/TiO_2_ NPs nanowires. The use of three different calcination temperatures, specifically 400 °C, 500 °C, and 600 °C, degraded the organic parts of hybrid fibrous mats, which were subjected to the electrospinning process. This contributed to the acquisition of amorphous nanowires of SiO_2_ and TiO_2_ regardless of the temperature applied (see Figure 3 and Figure 4).

The diffractive spectrum of electrons in the form of diffractive reflexions shaped like fuzzy orbs was obtained for silicon oxide nanowires and produced as a result of calcination of PVP/TEOS nanofibers at a temperature of 600 °C as well as composite PVP/TEOS nanofibers that contained nanoparticles of silicon oxide, which is evidence of their amorphous structure (see Figure 3c,f). The obtained SiO_2_ materials can be considered as amorphous nanowires with a diameter of approximately 100 nm and a length of no more than 1 μm. The analysis of the morphology of the studied hybrid ceramic SiO_2_/SiO_2_ NPs nanostructures, which was conducted on the basis of the registered TEM images, showed an even dispersion of the applied reinforcing phase in the entire volume of single silica nanowires (see Figure 3d,e). 

The analysis of the structure and morphology of a single TiO_2_ nanostructure, which is visible in Figure 4a,b, unambiguously shows that the obtained structure can be defined as a one-dimensional structure in the form of a nanowire with a diameter of 500 nm and a length of approximately 3 μm. Moreover, the ratio of the diameter to the length of the obtained one-dimensional structure of TiO_2_ allows the classification of the produced nanomaterial as a nanowire. The diffractive spectrum of electrons was obtained for a single TiO_2_ nanowire in the form of diffractive reflexions shaped like fuzzy orbs and created as a result of the dispersion of the electron beam. This is evidence of their amorphous structure (see Figure 4c). The addition of titanium oxide nanoparticles to the spinning solution of PVP/TNBT/EtOH/AcOH allowed to obtain composite PVP/TEOS/TiO_2_ NPs nanowires, which allowed us to obtain bimodal ceramic TiO/TiO_2_ NPs nanowires as a result of the conducted calcination process (see Figure 4d,e). The analysis of the structure and morphology of the produced titanium oxide nanowires reinforced with TiO_2_ nanoparticles showed an even dispersion of the reinforcing phase in the entire volume of single bimodal, semi-conductive nanostructures (see Figure 3). The electronogram obtained for the bimodal TiO_2_ nanowires shows a collection of diffraction images from the amorphous matrix of titanium oxide as well as diffractive reflexions from plains with the following Miller’s indexes: (125), (121), (220), (020) and (011), corresponding to the structure of nanocrystalline anatase, which was displayed by the used titanium oxide nanoparticles and played the role of the reinforcing phase.

### 3.2. FTIR Analysis

**Figure 5:**
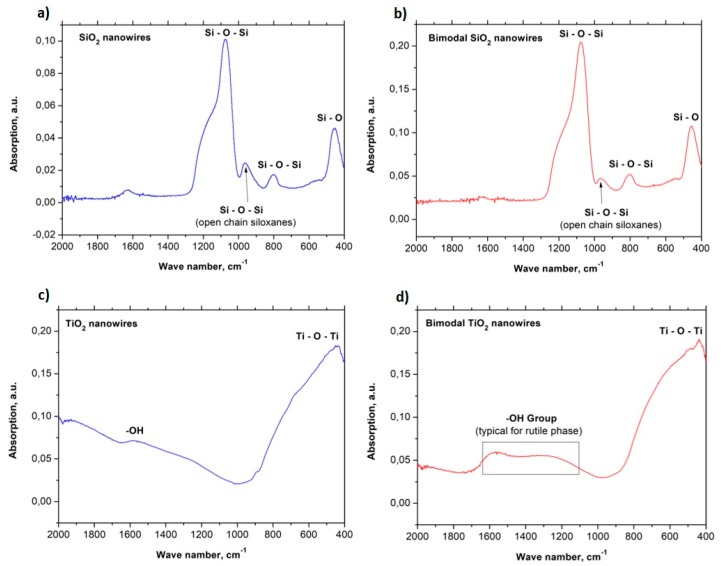
FTIR spectra for: (**a**) SiO_2_ nanowires, (**b**) bimodal SiO_2_ nanowires, (**c**) TiO_2_ nanowires, (**d**) bimodal TiO_2_ nanowires.

The absorbance spectra in the function of the wavenumber in the range of 2000 to 400 cm^−1^ graphs were plotted for the all groups of the obtained ceramic and bimodal one-dimensional nanostructures with some characteristic peaks for individual vibration molecules or functional groups (see Figure 5). FTIR examination of pure one-dimensional silica structures reveals two bond types occurring in the chemical structure of SiO_2_ nanowires including Si-O-Si and Si-O, which correspond to the following values of the wavenumber: 451, 802, 964, and 1072 cm^−1^, respectively. This coincides with the results obtained in the works [40,41]. In addition, the peaks obtained in the FTIR spectrum, which were obtained for bimodal SiO_2_/SiO_2_ nanowires, correspond to the bonds present in the chemical structure of the reinforcing phase. 451 cm^−1^ corresponds to the rocking vibration type of Si-O while the peaks registered for frequencies of 802, 964, and 1076 cm^−1^ correspond to the stretching bond type of Si-O-Si [42] (Figure 5a,b). Moreover, Si-O-Si bonds are responsible for making SiO_2_ molecules. In the case of titanium oxide nanowires, the FTIR spectra show the Ti-O-Ti bond type, which corresponds to the wavenumber value of 440 cm^−1^. In addition, in the range of the wavenumber with a value of about 1600 cm^−1^, OH groups were observed, which correspond with the spectra obtained in the work [43] (see Figure 5c).

For bimodal TiO_2_ nanowires, absorption peaks were also observed, which correspond to Ti-O-Ti bonds as well as a significant extension of the spectrum indicative of OH groups (see Figure 5d). This phenomenon most likely results from the presence of evenly dispersed TiO_2_ nanoparticles in the amorphous matrix of titanium oxide nanowires. The increase of radiation absorption resulting from the presence of oxide particles can be explained by the increase of the specific surface area of the obtained nanostructures in comparison to TiO_2_ nanowires that do not contain nanoparticles inside of them or on their surface [42] as well as the crystal structure of rutile of the particles used [35]. The wide range of absorption, which corresponds to OH groups, is characteristic for TiO_2_ in the rutile phase and results from the bending vibration of chemically adsorbed water [43,44]. The results obtained for the FTIR analysis of the produced ceramic nanowires confirm the research results obtained with the use of a high-resolution transmission electron microscope, which proved the existence of crystal TiO_2_ nanoparticles in the amorphous matrix of titanium oxide nanowires. 

### 3.3. SEM Analysis

In order to analyze the morphology and structure of the produced hybrid and composite nanofibers as well as the obtained oxide nanowires, imaging of the topography of the tested materials was carried out using a scanning electron microscope. Analysis of the morphology and structure of PVP/TEOS hybrid nanofibers obtained from a PVP/TEOS/EtoH/AcOH solution with 10% share of polymer by weight showed that the obtained fibers constituting the starting material to produce silicon oxide nanowires were free of structural defects and had constant values of diameters along all their length (see Figure 5a).

**Figure 6:**
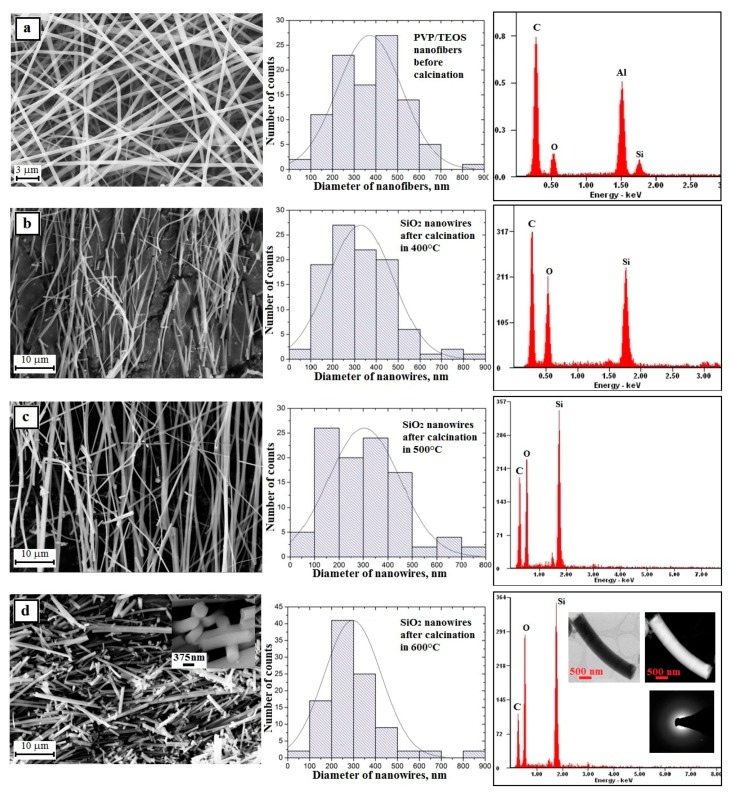
Left: SEM image of the topography on the surface of the formed fibrous composite mats, obtained nanowires; Middle: histograms presenting the distribution of hundredfold measurement of the diameter of randomly selected: (**a**) nanofibers obtained from PVP/TEOS/H_2_O/AcOH/EtOH solution, (**b**–**d**) SiO_2_ nanowires after calcination in 400 °C, 500 °C, and 600 °C; Right: obtained EDS spectra (peaks of Al derive from the substrate on which nanofibers were deposited while Au and Pd derive from the conductive layer sputtered onto the produce nanostructures) from the entire area shown in the SEM images and TEM images for SiO_2_ nanowires obtained in 600 °C including an image in a light and dark area with diffraction image from single nanowires.

A hundred-fold measurement of the diameters of the obtained PVP hybrid nanofibers containing TEOS precursor particles showed that the measured values ranged from 60 nm to 830 nm with the most frequent diameters ranging from 400 nm to 500 nm, which accounted for 27% of all measured diameters. In addition, for this sample, the average diameter value was 371 nm (Figure 6a, histogram).

**Figure 7:**
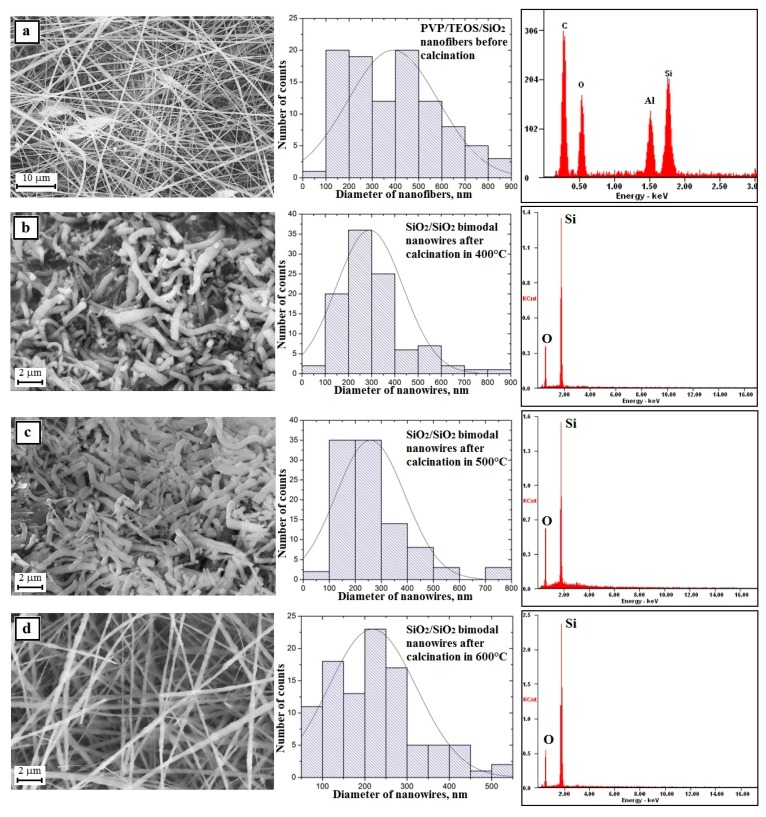
Left: SEM image of the topography on the surface of the formed fibrous composite mats, obtained nanowires; Middle: histograms presenting the distribution of hundredfold measurement of the diameter of randomly selected: (**a**) nanofibers obtained from PVP/TEOS/SiO_2_/H_2_O/AcOH/EtOH solution, (**b**–**d**) bimodal SiO_2_/SiO_2_ nanowires after calcination in 400, 500 and 600 °C; Right: obtained EDS spectra (peaks of Al derive from the substrate on which nanofibers were deposited while Au and Pd derive from the conductive layer sputtered onto the produce nanostructures) from the entire area shown in the SEM images.

The next stage of the research consisted in calcination at three different temperatures of the obtained PVP/TEOS hybrid fibrous mats for three hours. This led to clean and defect-free structural nanowires of silicon oxide. This was confirmed by the analysis of the EDS spectra obtained for these materials (Figure 6b–d—EDS spectra). Regardless of the temperature used during the calcination process, the obtained one-dimensional SiO_2_ nanostructures were characterized by a lack of structural defects and a hundred-fold diameter measurement of the obtained nanowires showed that the measured diameters were in close ranges of the nanoscale. The hybrid calcinations, by successively applying temperatures of 500 °C and 600 °C, were allowed to produce SiO_2_ nanowires with diameters from 80 nm to 750 nm and from 80 nm to 830 nm (Figure 6b–d—histograms). The temperature of polymer matrix degradation was increased in the calcined nanofibers where the average diameter value decreased and was 328 nm, 304 nm, and 296 nm, respectively. This fact indicates the possibility of controlling the morphology of the produced nanowires by changing the calcination temperature and obtaining amorphous SiO_2_ nanostructures in each case (Figure 5d—TEM images and diffraction spectrum). The addition of up to 10% PVP/TEOS/EtoH/AcOH spinning solution of SiO_2_ nanoparticles resulted in an increase in the diameters of the obtained PVP/TEOS/SiO_2_ hybrid composite nanofibers by about 20 nm compared to fibers containing no silica nano powder as well as the formation of structural defects in the beads concentrating the agglomerates used as the nano filler (Figure 7a—picture SEM and histogram). Subjecting the obtained composite mat to a calcination process, at each temperature, allowed us to obtain bimodal SiO_2_ nanowires containing SiO_2_ nanoparticles within their volume and on their surface (Figure 7b–d and EDS spectra). In the case of bimodal silicon oxide nanowires, in contrast to the pure SiO_2_ nanostructures obtained from the PVP/TEOS solution, the temperature increase used during the calcination process led the range of recorded diameters of the tested one-dimensional nanostructures to decrease. However, after calcination at 400 °C, the measured diameters of SiO_2_ did not exceed 900 nm.

The application of subsequent temperatures allowed us to obtain nanowires with diameters not exceeding 800 nm (500 °C) and 550 nm (600 °C) (Figure 7b–d—histograms). This fact indicates the possibility of controlling the morphology of the produced nanowires by changing the calcination temperature and obtaining amorphous SiO_2_ nanostructures in each case (Figure 6d—TEM images and diffraction spectrum) and adding the same phase of the nano powder to the spinning solution.

**Figure 8:**
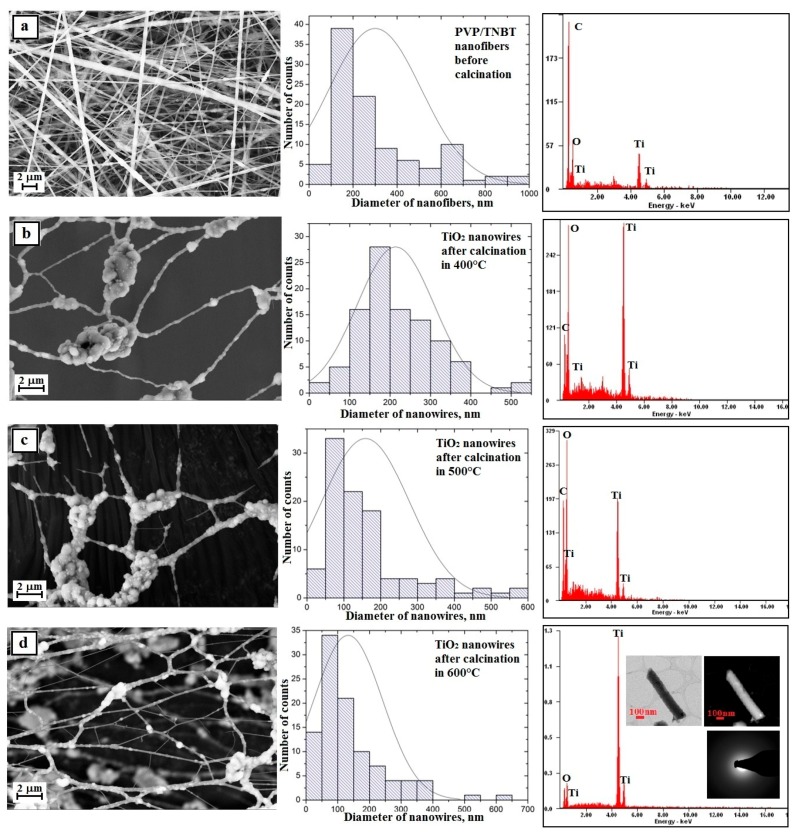
Left: SEM image of the topography on the surface of the formed fibrous composite mats, obtained nanowires; Middle: histograms presenting the distribution of hundredfold measurement of the diameter of randomly selected: (**a**) nanofibers obtained from PVP/TNBT/AcOH/EtOH solution, (**b**–**d**) TiO_2_ nanowires after calcination in 400 °C, 500 °C, and 600 °C; Right: obtained EDS spectra (peaks of Al derive from the substrate on which nanofibers were deposited while Au and Pd derive from the conductive layer sputtered onto the produce nanostructures) from the entire area shown in the SEM images and TEM images for TiO_2_ nanowires obtained in 600 °C an image in a light and dark area with diffraction image from single nanowires.

The analysis of the morphology and structure of the hybrid nanofibers of PVP/TNBT precursor molecules, which were obtained from PVP/TNBT/EtoH/AcOH solution with 10% polymer concentration (by weight) and showed that these fibers have defects in the form of the so-called beads that were probably formed by the hydrolysis and condensation of the precursor particles already at the stage of the spinning solution (see Figure 8a).

A hundred-fold measurement of the diameters of the obtained PVP/TNBT hybrid nanofibers showed that the measured diameters ranged from 50 nm to 1000 nm with the most frequent diameters ranging from 100 nm to 200 nm, which accounted for 38% of all measured diameters for these samples where the average value of the nanofibers studied was 278 nm (Figure 8a—histogram).

Subjecting the obtained PVP/TNBT fibrous mat to a thermal treatment at temperatures of 400 °C, 500 °C, and 600 °C allowed us to obtain, in each case, pure titanium oxide nanostructures (see Figure 6b–d), which form a characteristic grid with characteristic knot-shaped connections and create a residue on the beads resulting from the precursor’s hydrolysis and condensation. This was confirmed by the analysis of the EDS spectra obtained for these materials (Figure 8b–d—EDS spectra). In order to further describe the results of the analyses, we will define the obtained TiO_2_ nanostructures as nanowires and the description will concern agglomerates of particles produced as a result of the conducted calcination process excluding the agglomerates themselves.

Research on the morphology of the obtained TiO_2_ nanowires created using the SEM microscope showed that the obtained one-dimensional ceramic nanostructures were characterized by smaller diameters of individual nanowires and compared to hybrid fibers used as the starting material for their production. Calcination of fibrous PVP/TNBT mats at high temperatures for 3 h allowed the removal of the organic phase from the fibers and the formation of titanium oxide nanowires whose measured diameters ranged from 40 nm to 650 nm for a sample annealed at 400 °C. Nanowires with diameters of 100–200 nm were the largest group and amounted to 28% (Figure 8b—histogram). The measured diameters of the TiO_2_ nanowires obtained by calcination at 500 °C were in the range of 80 to 600 nm. The largest group constituting 33% of all nanowires of this sample was comprised of nanowires with diameters of 50–100 nm (Figure 7c—histogram). The smallest diameter values were recorded for titanium oxide nanowires resulting from the calcination process of PVP/TNBT hybrid nanofibres at 600 °C. For this sample, the generated nanostructures were characterized by values of measured diameters in the range from 30 to 610 nm (Figure 8d—histogram). In addition, a hundred-fold measurement of randomly chosen nanowires of this sample showed that the largest group of 34% were one-dimensional structures with diameters ranging from 50 nm to 100 nm. The analysis of the average values of diameters of the produced TiO_2_ nanowires, which were successively 214 nm, 159 nm, and 153 nm after calcination at temperatures of 400 °C, 500 °C, and 600 °C, unambiguously shows a significant influence of temperature on the morphology of the manufactured one-dimensional titanium oxide nanostructures.

**Figure 9:**
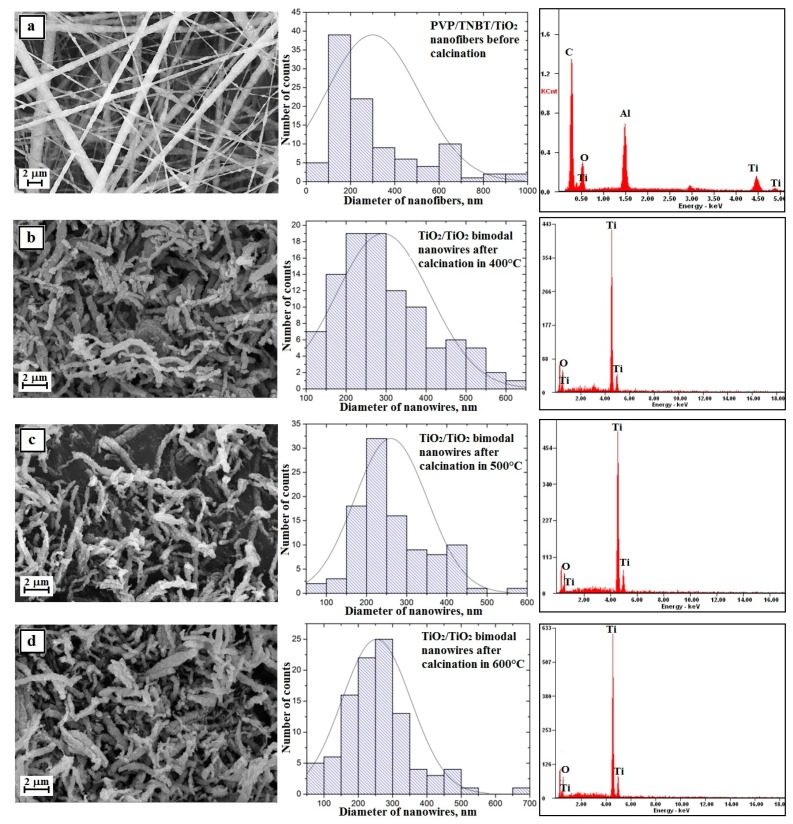
Left: SEM image of the topography on the surface of the formed fibrous composite mats and obtained nanowires; Middle: histograms presenting the distribution of hundredfold measurement of the diameter of randomly selected: (**a**) nanofibers obtained from PVP/TNBT/TiO_2_/AcOH/EtOH solution, (**b**–**d**) bimodal TiO_2_/TiO_2_ nanowires after calcination in 400 °C, 500 °C, and 600 °C; Right: obtained EDS spectra (peaks of Al derive from the substrate on which nanofibres were deposited, while Au and Pd derive from the conductive layer sputtered onto the produce nanostructures) from the entire area shown in the SEM images and TEM images for TiO_2_ nanowires obtained in 600 °C an image in a light and dark area with diffraction image from single nanowires.

The analysis of the SEM image of the surface topography of a fibrous mat made of PVP/TNBT hybrid composite nanofibers containing titanium dioxide nanoparticles with a 25% concentration by mass indicates that, unlike nanofibers made from a solution containing no TiO_2_ nanoparticles, the obtained one-dimensional PVP/TNBT/TiO_2_ structures were free of structural defects in the form of visible nanoparticle agglomerates of the used nano filler (see Figure 9a).

Structural defects in the form of beads resulting from the phenomenon of hydrolysis and condensation of molecules of the used TNBT precursor were also not registered while the average diameter value of the obtained nanofibers was 300 nm, which clearly demonstrates the positive effect of the presence of nanoparticles of titanium oxide used in the spinning solution on the morphology and structure of the obtained hybrid composite nanofibers of PVP/TNBT/TiO_2_.

Subjecting the obtained PVP/TEOS/TiO_2_ fibrous mats to the calcination process at temperatures of 400 °C, 500 °C, and 600 °C allowed to obtain one-dimensional bimodal nanostructures of pure titanium oxide (Figure 9b–d—EDS spectra). Bimodal TiO_2_/TiO_2_ nanowires obtained by the calcination of a fibrous mat at 400 °C were characterized by diameters ranging from 100 nm to 700 nm. The most numerous group of 38% consisted of nanowires with diameters ranging from 200–300 nm (see Figure 9b). An increase in the calcination temperature to 500 °C caused a slight change in the recorded diameters of the obtained nanowires. In this case, the diameters were in the range from 90 nm to 700 nm and the most numerous group of 32% consisted of TiO_2_/TiO_2_ nanowires with diameters in the range of 200–250 nm (see Figure 9c). The use of the highest calcination temperature of 600 °C resulted in similar results to those obtained after the calcination process of the hybrid nanofibrous PVP/TEOS/TiO_2_ mat at 500 °C. The resulting bimodal TiO_2_/TiO_2_ nanowires were characterized by diameters ranging from 80 nm to 700 nm with the most frequently occurring diameters ranging from 250 nm to 300 nm (see Figure 9d). The analysis of the morphology of the produced bimodal TiO_2_ nanowires showed a decrease in the average diameters of one-dimensional nanostructures along with a temperature increase during the calcination process. The use of the calcination process for three hours at temperatures of 400 °C, 500 °C, and 600 °C, successively, contributed to the production of bimodal one-dimensional TiO_2_/TiO_2_ nanostructures with diameters of 293 nm, 261 nm, and 253 nm.

The comparison of the surface topography of the produced TiO_2_ nanowires and bimodal TiO_2_/TiO_2_ nanowires indicates a significant effect of the applied ceramic nanoparticles on the surface structure of the obtained one-dimensional nanostructures (see Figure 8). Regardless of the calcination temperature used, the surface area of the obtained one-dimensional bimodal titanium dioxide nanomaterials, compared to nanowires obtained from the PVP/TNBT/EtOH solution, was clearly uneven and was characterized by numerous “frayings”. The mechanism of the occurrence of uneven, fuzzy surface of the produced bimodal TiO_2_/TiO_2_ nanostructures, which accompanied the share of TiO_2_ nanoparticles in the spinning solution, has a significant impact on the increase in the specific surface area of the obtained titanium oxide nanostructures. This results in a significant extension of the application possibilities of this type of materials or used devices built on the basis of TiO_2_ nanowires. Increasing the contact surface of the produced one-dimensional nanostructures of titanium oxide with catalysts will significantly affect the rate of photo catalysis, which is desirable in the case of self-cleaning surfaces. In addition, the use of TiO_2_ nanowires with the observed surface morphology may potentially result in an increase in the efficiency of the new generation of photovoltaic cells through the increase of the contact surface of semi-conductive bimodal, one-dimensional TiO_2_/TiO_2_ nanostructures with dye particles.

### 3.4. Optical Investigations

**Figure 10:**
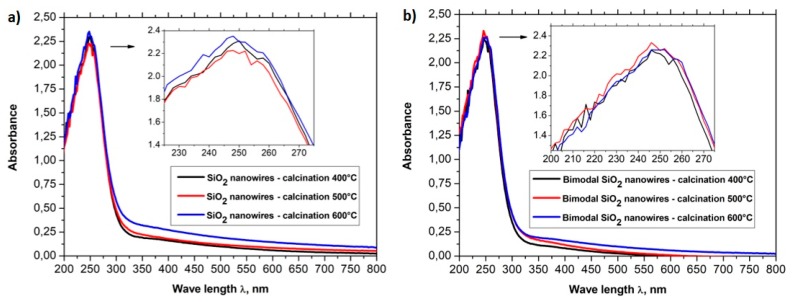
UV-Vis spectra obtained for (**a**) SiO_2_ nanowires and (**b**) bimodal SiO_2_ nanowires.

In order to analyze the optical properties of the produced one-dimensional, ceramic nanomaterials, the spectra of absorption in the function of wavelength obtained using a UV-Vis spectrometer were registered for all six groups of nanowires (see Figure 10a,b). The absorption spectra in the function of wavelength registered for SiO_2_ nanowires as well as for bimodal SiO_2_/SiO_2_ nanowires showed that the addition of silica nanoparticles to the spinning solution did not contribute to a change in the optical properties of the one-dimensional nanostructures of silicon oxide (see Figure 10a ,b). The absorption dependencies in the function of wavelength obtained for SiO_2_ nanowires and bimodal SiO_2_/SiO_2_ nanowires showed the presence of a sharp absorption edge for wavelengths of approximately 300 nm while the absorption maxima corresponded with wavelengths of approximately 250 nm.

The spectral characteristics that were registered for one-dimensional nanowires of titanium oxide showed the presence of a sharp absorption edge in the region of close ultraviolet. The presence of the sharp absorption edge concerned wavelengths of approximately 325 nm. Additionally, it was shown that with an increase of the temperature of calcination of hybrid PVP/TNBT nanofibers, which constitute the starting material for the production of one-dimensional nanostructures of titanium oxide, the obtained TiO_2_ nanowires were characterized by a constant maximum of absorption for wavelengths of 248 nm as well as a linear decrease of the level of absorption of electromagnetic radiation from 2.42 nm for nanowires obtained at a temperature of 400 °C to 2.26 nm for nanowires obtained during the process of calcination at a temperature of 600 °C (see Figure 10a).

For bimodal TiO_2_/TiO_2_ nanowires (see Figure 10b), the obtained spectrum showed the presence of strong absorption in the ultraviolet range where in the rage of close ultraviolet as well as in the rage of visible light, the level of absorption for bimodal nanostructures of titanium oxide obtained by using temperatures in the range of 400–600 °C during the calcination process, which reached higher values than those registered for nanowires of titanium oxide that did not contain TiO_2_ particles. In the rage of visible light, the level of absorption of electromagnetic radiation increased from a value of 0.5 to a value of approx. 1.1 while, in the range of close ultraviolet, the level of absorption for bimodal TiO_2_/TiO_2_ reached a value of 3.5. The results of the research on the effects of bimodal one-dimensional nanostructures of titanium oxide with electromagnetic radiation unambiguously point to the improved optical properties of such structures in comparison to conventional TiO_2_ nanowires. A more effective absorption of ultraviolet radiation can contribute to an increased speed of the photocatalytic reaction that can be of great significance in the case of TiO_2_, which is considered a material with increased photocatalytic properties and is used in such application as self-cleaning, superhydrophilic, antibacterial, antistatic, and deodorizing coatings. Moreover, this type of materials can constitute a more effective alternative for thin semi-conductive coatings currently used for the production of dye-sensitized solar cells due to a much larger specific surface area in relation to the homogeneous layers obtained when using the spin-coating process.

**Figure 11:**
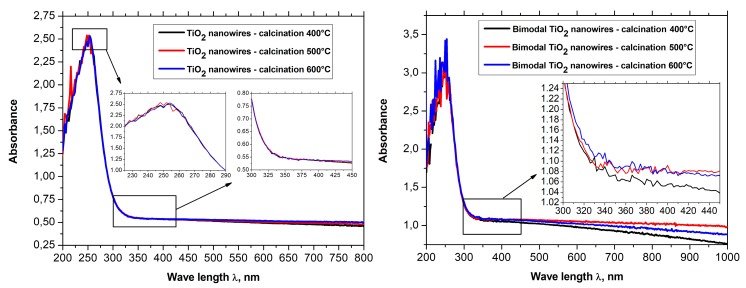
UV-Vis spectra obtained for (**a**) TiO_2_ nanowires and (**b**) bimodal TiO_2_ nanowires.

**Figure 12:**
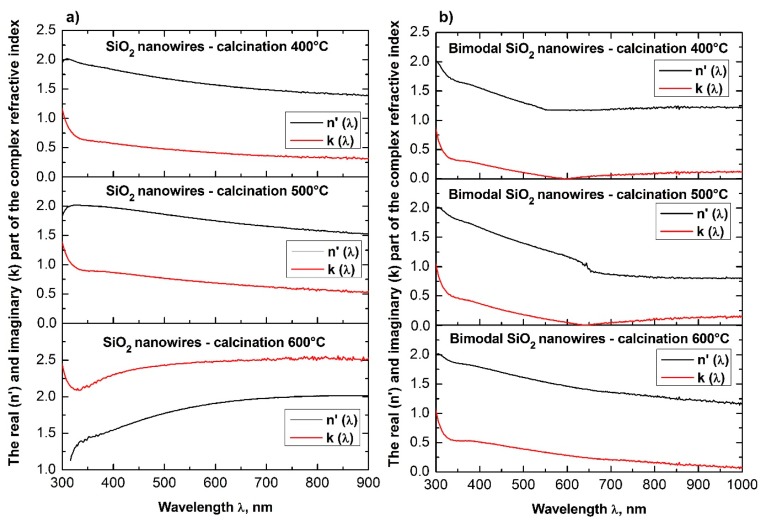
Dependences of the real *n*′(*λ*) (**a**) and imaginary *k*(*λ*) parts (**b**) of the refractive index determined for the produces SiO_2_ ceramic nanowires and bimodal SiO_2_/SiO_2_ nanowires.

**Figure 13:**
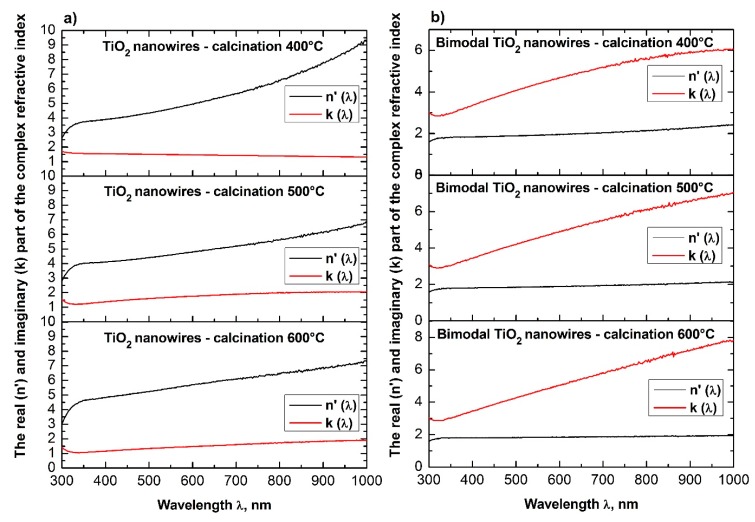
Dependences on the real *n*′(*λ*) (**a**) and imaginary *k*(*λ*) parts (**b**) of the refractive index determined for the produced TiO_2_ ceramic nanowires and bimodal TiO_2_/TiO_2_ nanowires.

**Figure 14:**
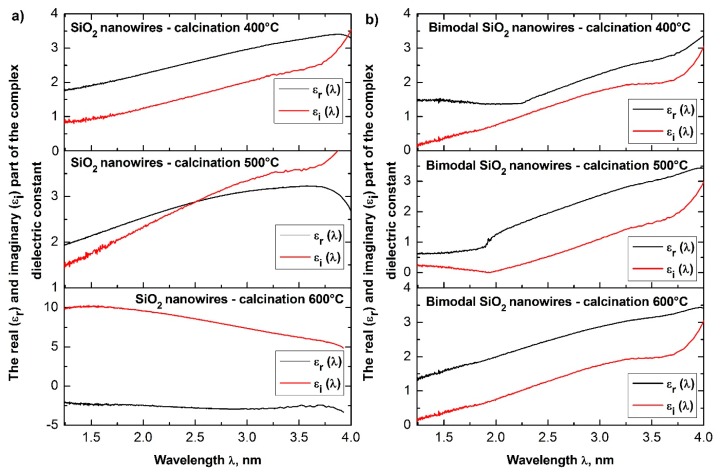
Dependences of the real *ε_r_*(*λ*) (**a**) and imaginary *ε_i_*(*λ*) parts (**b**) of the dielectric constant for the produced SiO_2_ ceramic nanowires and bimodal SiO_2_/SiO_2_ nanowires.

**Figure 15:**
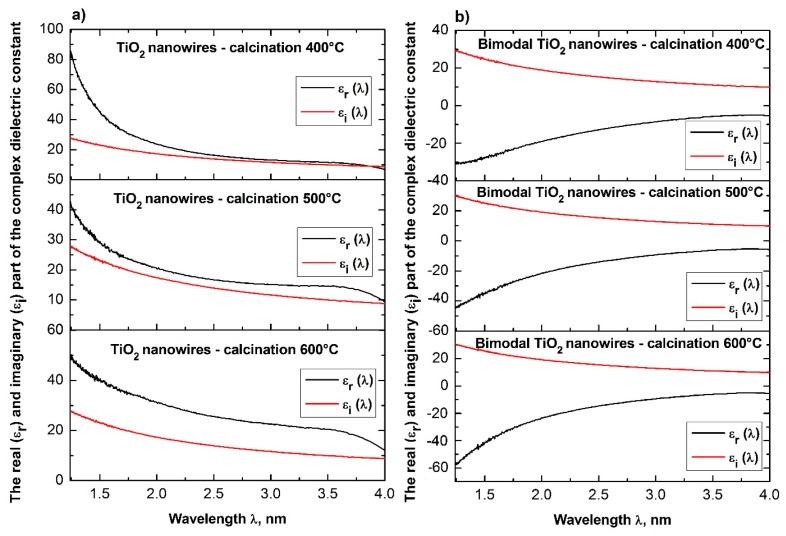
Dependences of the real *ε_r_*(*λ*) (**a**) and imaginary *ε_i_*(*λ*) parts (**b**) of the complex dielectric constant for the produced TiO_2_ ceramic nanowires and bimodal TiO_2_/TiO_2_ nanowires.

In order to analyze the optical properties of the produced single-dimensional ceramic nanomaterials, for all four groups of nanowires, the spectra of absorbance as a function of wavelength were obtained using a UV-Vis spectrometer. Based on the obtained dependences *A*(*λ*) and the equations derived in the Theory section, the dependencies of the real *n*′(*λ*) and imaginary *k*(*λ*) part of the refractive index, real and complex dielectric permittivity *ε_r_*(*λ*), *ε_i_*(*λ*), and complex values of the refractive index, dielectric permittivity and energy gap width values for the produced ceramic SiO_2_, TiO_2_ and bimodal nanowires SiO_2_/SiO_2_, and TiO_2_/TiO_2_ (Figure 7, Figure 8, Figure 9, Figure 10, Figure 11, Figure 12, Figure 13, Figure 14 and Figure 15) were obtained.

The analysis of the complex refractive index for the produced SiO_2_ ceramic nanowires indicates that subjecting the thin fibrous mat containing nanofibers obtained from the solution of PVP/TEOS/AcOH/EtOH to the calcination process at 400 °C for 3 h contributed to obtaining silicon oxide nanowires, which were characterized by the smallest determined value of the refractive index among all the produced and tested one-dimensional ceramic structures (see Table 1).

This value corresponds to the refractive index for SiO_2_ presented in [45], which clearly indicates the accurateness both of the process of manufacturing silicon oxide nanowires and the validity of the carried out of the theoretical considerations and experimental analyses.

Annealing of hybrid nanofibers of PVP/TEOS at 500 °C increased the obtained value of the refractive index of 0.02 relative to the refractive index of SiO_2_ nanowires received at the lowest used temperature.

The use of the highest temperature during the calcination process of 600 °C resulted in obtaining silicon oxide nanowires characterized by the highest optical density of which refractive indices reached the value of 1.60. The obtained value of complex refractive index of 1.46 for SiO_2_ ceramic nanowires produced during the calcination of hybrid PVP/TEOS nanofibers at a temperature of 400 °C corresponds to the refractive index for silicon oxide presented by Malitson in Reference [45], which clearly indicates the accuracy of the manufacturing process of silicon oxide nanowires and the validity of the theoretical considerations and experimental analyses.

Furthermore, the increase of the complex refractive index values of the SiO_2_ nanowires obtained at different calcination temperatures (400 °C to 600 °C) is probably related to the morphology of the tested ceramic nanostructures.

The analyses indicate the influence of the diameter of the tested silica nanowires on their optical properties. During the acquisition process of SiO_2_ nanowires characterized by the same structure and chemical composition, it is possible to manipulate the optical density values of the used ceramic nanostructures by changing the diameters of individual nanowires.

The addition of silicon oxide nano powder to the PVP/TEOS/AcOH/EtOH spinning solution allowed to obtain bimodal, amorphous one-dimensional SiO_2_ nanostructures that were characterized by higher optical densities were compared to the optical density of SiO_2_ nanowires obtained from a solution containing no nanoparticles in the form of silica nanoparticles. Bimodal SiO_2_/SiO_2_ nanowires obtained by the calcination of composite PVP/TEOS/SiO_2_ nanofibers at 400 °C for 3 h were characterized by the same values of complex refractive index light of 1.60, which had pure silicon oxide nanowires produced at a calcination temperature of 600 °C.

The application of higher temperatures during the calcination process of PVP/TEOS/SiO_2_ composite nanofibers allowed to maintain one-dimensional SiO_2_/SiO_2_ ceramic nanostructures with complex refractive index values of 1.71 in the case of nanowires obtained by calcination at 500 °C as well as 1.73 for nanowires obtained at 600 °C.

This fact demonstrates the significant influence of the presence of a nano filler on the optical properties of the produced bimodal, monomaterial ceramic nanomaterials, and the potential to control their properties by changing the mass concentration of silica nanoparticles in the spinning solution.

The analysis of the optical properties of one-dimensional titanium oxide nanostructures obtained due to calcination of thin fibrous PVP/TNBT hybrid mats showed a decrease in the value of the refractive index of TiO_2_ ceramic nanowires accompanying a temperature increase during the calcination process (see Table 1). Titanium oxide nanowires obtained as a result of calcination at 400 °C were characterized by the highest optical density of all the obtained one-dimensional TiO_2_ nanostructures. In this case, the maximum refractive index amount to 2.62. The application of calcination temperatures of 500 °C enabled the formation of nanowires of titanium oxide with a refractive index of 2.57. The use of a calcination temperature of 600 °C allowed to obtain TiO_2_ nanowires with the lowest registered optical density—the value of their complex refractive index was 2.49. The obtained results of the complex refractive index of the produced TiO_2_ ceramic nanowires coincide with the results presented in References [46,47,48,49], which confirm the correctness of the theoretical and experimental analyses.

Bimodal TiO_2_/TiO_2_ nanowires obtained by the calcination of composite PVP/TNBT nanofibers containing 25% (wt %) of titanium oxide nanoparticles showed an opposite trend in the range of recorded electromagnetic radiation, which was compared to pure one-dimensional TiO_2_ nanostructures. The optical density of the obtained bimodal nanowires of titanium oxide increased as a result of the higher temperature of calcination. Bimodal TiO_2_/TiO_2_ nanostructures obtained at 400 °C were characterized by the same value of complex refractive index as TiO_2_ nanowires obtained at 600 °C. A further increase in temperature to 500 °C and 600 °C during the calcinations process of the composite PVP/TNBT/TiO_2_ nanofibers resulted in obtaining one-dimensional bimodal TiO_2_/TiO_2_ nanostructures with n coefficients of 2.52 and 2.57, respectively. This phenomenon is probably due to the phase composition of the applied titanium oxide nano powder containing both the rutile phase and the anatase phase.

The dependence of dielectric permeability as a function of the radiation frequency incident on the sample can be presented in the following form [50].

(19) $$\varepsilon =1+\frac{\mu e^{2}}{m\varepsilon_{0}}\left[\frac{\omega_{0}^{2}-\omega^{2}}{{\left(\omega_{0}^{2}-\omega^{2}\right)}^{2}+\gamma^{2}\omega^{2}}-i\frac{\gamma \omega }{{\left(\omega_{0}^{2}-\omega^{2}\right)}^{2}+\gamma^{2}\omega^{2}}\right]$$

where *μ* is the concentration of atoms in the sample, *e* and *m* is the charge and mass of the electron, *ε*_0_ is the electric permeability of the vacuum, *γ* is the damping factor, and *ω*_0_ and *ω* are the frequencies of electrons, own vibrations, and radiation. From the above Equation (19), it follows that the dielectric constant is a complex value while its real and imaginary part can be written as the equations below.

(20) $$\varepsilon \prime =1+\frac{ne^{2}}{m\varepsilon_{0}}\frac{\omega_{0}^{2}-\omega^{2}}{{\left(\omega_{0}^{2}-\omega^{2}\right)}^{2}+\gamma^{2}\omega^{2}}$$

(21) $$\varepsilon \prime \prime =\frac{ne^{2}}{m\varepsilon_{0}}\frac{\omega_{0}^{2}-\omega^{2}}{{\left(\omega_{0}^{2}-\omega^{2}\right)}^{2}+\gamma^{2}\omega^{2}}$$

Using the relation binding the index of refraction and the dielectric constant (9), the expressions on the real and imaginary part of optical permeability take the form below [50].

(22) n′={12[ε′+(ε′2+ε″2)]12}12

(23) n″={12[−ε′+(ε′2+ε″2)]12}12

The above equations show that with the increase of the refractive index of the tested medium, its dielectric permittivity increases. The analyses of the determined complex dielectric permittivity values of the produced ceramic nanowires of SiO_2_ and TiO_2_ as well as bimodal nanowires of SiO_2_/SiO_2_ and TiO_2_/TiO_2_ (see Table 1), which were conducted on the basis of the presented theoretical considerations and recorded spectra of absorbance as a function of the wavelength and coincide with the theoretical assumptions resulting from the dependence *n* = *ε*^1/2^.

The analysis of the width of energy gaps in the studied amorphous ceramic nanowires unambiguously indicated a significant influence of the applied temperature during the calcination process and the presence of nanoparticles of the nano-filler in the obtained composites on the energy barrier between the conduction band and the valence band of the studied nanomaterials (see Table 1). The use of a temperature in the range of 400 °C to 600 °C during the annealing of hybrid PVP/TEOS nanofiber resulted in the formation of SiO_2_ nanowires characterized by values of the energy gap of 4.19, 4.31, and 4.40 eV, respectively. The 25% mass presence of silica nanoparticles in the spinning solution resulted in the opposite tendency of changing the values of the energy band gaps characteristic for bimodal SiO_2_/SiO_2_ nanowires, which resulted from the use of higher temperatures during the calcination process. In this case, the ever-higher temperature of composite PVP/TEOS/SiO_2_ nanofibers heating contributed to a decrease in the determined values of energy band gaps from 5.5 eV to 4.29 eV. The tendency of decreasing values of the indicated energy gaps, which accompanies the increase of temperature during the calcination process, is in accordance with the results presented in papers on semi-conductive ceramic nanowires [51,52] in which the same calcination temperatures of 400 °C, 500 °C, and 600 °C were applied in order to obtain ceramic nanostructures. A decrease of the values of the indicated energy gaps, which accompanies the use of increasing calcination temperatures, most likely results from the morphology of the obtained nanostructures. It can be assumed that the use of increasing calcination temperatures of the produced bimodal SiO_2_ nanowires cause a decrease of the obtained nanostructures and is associated with obtaining nanomaterials with decreasing values of the energy gap.

In addition, the calcination processes applied to hybrid fibrous PVP/TEOS mats and hybrid composite fibrous PVP/TEOS mats containing SiO_2_ nanoparticles resulted in the formation of SiO_2_ nanowires obtained by the sol-gel and electrospinning processes from the solution, which causes the formation of SiO_2_ nanowires with energy band gaps values ranging from 4.19 eV to 4.5 eV, which is more than twice as good as the currently obtained amorphous silica structures [53].

The experimentally determined values of energy breaks in the produced SiO_2_ nanowires clearly indicate a broad spectrum of future applications including the electronics industry for building computer chips and solar batteries [54].

Similar relationships between the applied temperature and the presence of nanoparticles in the spinning solution were recorded for ceramic TiO_2_ nanowires and bimodal TiO_2_/TiO_2_ nanowires. In this case, similarly as in the case of bimodal nanowires of silicon oxide, the addition of nanoparticles to the spinning solution resulted in obtaining bimodal TiO_2_/TiO_2_ nanowires of which values of energy gap decreased when using increasing temperatures during the calcination process of composite fibers. The decrease of the indicated values of the energy gap for bimodal nanowires of titanium oxide most likely results from the decrease of the diameters of those structures, which can be observed when applying increasing annealing temperatures. The obtained values of energy breaks oscillating around 3.7 eV can be caused by direct electron transitions from the valence band to the conduction band where the smallest determined *E_g_* value of 3.30 eV corresponds to the intermediate electron transitions between the energy bands.

## 4. Conclusions

Using the sol-gel and electrospinning method, the aim of this work was to create and examine the morphology, structure, and optical properties of four types of one-dimensional ceramic nanostructures, which included TiO_2_ nanowires, SiO_2_ nanowires, and bimodal TiO_2_ nanowires filled with TiO_2_ nanoparticles and bimodal SiO_2_ nanowires filled with SiO_2_ nanoparticles. Particularly noteworthy is the fact that the author presented a method of obtaining composite nanofibers without structural defects constituting the starting material for the production of bimodal ceramic nanowires containing nano-fillers in the form of ceramic nanoparticles with mass concentration relative to the polymer mass of up to 25%. It has been found that the use of nanoparticles with such a mass concentration at the stage of the spinning solution preparation allows to obtain bimodal nanowires with a much larger specific surface area than their nanofiller-free counterparts resulting from several times smaller diameters of individual nanostructures. In addition, it was shown that the use of nano-fillers in the form of nanoparticles of the same phase, which was the matrix of each type of nanowires and different calcination temperatures in the range 400–600 °C, has a significant effect on the optical properties of the obtained one-dimensional ceramic nanostructures that were in the ranges 1.46–2.62 for a complex refractive index, 2.13–6.86 for combined dielectric permeability, and 3.3–4.5 eV for the energy gap width. The presented results of the research on innovative bimodal one-dimensional nanostructures unambiguously indicate the potential for controlling both the morphology and the optical properties of the obtained nanomaterials, which increase the scope of their application capabilities.

## Figures and Tables

**Table 1:** Determined values of the complex refractive index, dielectric permittivity and energy gap width values obtained for the produced nanowires.

Parameter	SiO_2_			SiO_2_/SiO_2_			TiO_2_			TiO_2_/TiO_2_		
	*Calcination Temperature* [°C]			*Calcination Temperature* [°C]			*Calcination Temperature* [°C]			*Calcination Temperature* [°C]		
	*400*	*500*	*600*	*400*	*500*	*600*	*400*	*500*	*600*	*400*	*500*	*600*
*n*	1.46	1.48	1.60	1.60	1.71	1.73	2.62	2.57	2.49	2.49	2.52	2.57
*ε*	2.13	2.19	2.56	2.56	2.92	2.99	6.86	6.60	6.20	6.20	6.35	6.60
*E_g_* [eV]	4.19	4.31	4.40	4.50	4.39	4.29	3.73	3.83	3.88	3.58	3.42	3.30
